# Neuroborreliosis Presenting as Encephalitis: A Case Report

**DOI:** 10.7759/cureus.57882

**Published:** 2024-04-08

**Authors:** Sabina David Ruban, Nanna Skaarup Andersen, Alena Svatkova, Christian Philip Fischer

**Affiliations:** 1 Department of Infectious Diseases, Copenhagen University Hospital Amager and Hvidovre, Copenhagen, DNK; 2 Clinical Centre for Emerging and Vector-Borne Infections, Department of Clinical Microbiology, Odense University Hospital, Denmark, Odense, DNK; 3 Research Unit of Clinical Microbiology, University of Southern Denmark, Odense, DNK; 4 Danish Research Centre for Magnetic Resonance, Centre for Functional and Diagnostic Imaging and Research, Copenhagen University Hospital Amager and Hvidovre, Copenhagen, DNK; 5 Department of Radiology, Centre for Functional and Diagnostic Imaging and Research, Copenhagen University Hospital Amager and Hvidovre, Copenhagen, DNK

**Keywords:** lyme neuroborreliosis, mri images, vector borne diseases, acute encephalitis, borrelia burgdorferi infection

## Abstract

Infection with *Borrelia burgdorferi* spirochetes can cause Lyme neuroborreliosis (LNB). Neuroborreliosis presenting as encephalitis is a rare manifestation. We present a 72-year-old male patient hospitalized after three days of confusion and altered mental status. Initial computerized tomography (CT) and magnetic resonance imaging (MRI) of the brain were both unremarkable. Lumbar puncture showed an elevated number of white blood cells, elevated protein, and normal glucose levels in the cerebrospinal fluid (CSF), normal electroencephalogram (EEG), and negative tests for common microorganisms in the CSF. The patient received treatment with acyclovir and ceftriaxone. Lumbar puncture repeated on day 16 showed a decreasing number of white blood cells. A repeated MRI showed white matter edema, interpreted as encephalitis, while a repeated EEG showed signs of a non-specific cerebral lesion. The first lumbar puncture revealed intrathecal immunoglobulin M (IgM) antibodies against *Borrelia* and was positive for *Borrelia* DNA using real-time PCR, and the following lumbar puncture showed both IgM and IgG intrathecal antibody production. These results thus confirmed the diagnosis of Lyme *Borrelia *encephalitis. The patient improved clinically and was discharged after treatment with ceftriaxone for three weeks. Encephalitis due to LNB should be considered as a differential diagnosis in cases with unexplained neurological symptoms. Changes in MRI and/or EEG might occur late in the course of the disease, underlining the need for repeated tests in unresolved cases.

## Introduction

Lyme disease is caused by spirochetes within the *Borrelia burgdorferi* sensu lato complex and is transmitted by ticks. The clinical course of Lyme disease can vary from an acute localized skin lesion (erythema migrans) after a tick bite, to a disseminated stage with neurological, cardiac, skin, or articular involvement. Lyme neuroborreliosis (LNB) is defined as a neurological manifestation of the disease. Common symptoms of LNB include painful radiculitis, peripheral facial nerve palsy, and headache. Radiculitis can present as multiple radiculitis, mononeuritis multiplex, or plexitis [1].

Based on criteria from the European Federation of Neurological Societies (EFNS), the diagnosis of LNB requires 1) neurological symptoms, 2) cerebrospinal fluid (CSF) pleocytosis, and 3) *Borrelia burgdorferi*-specific antibodies produced intrathecally [2].

Both the central nervous system (CNS) and the peripheral nervous system (PNS) can be involved in LNB. Symptoms of encephalitis such as confusion or other signs of CNS involvement are rare.

Here, we report the case of encephalitis as a rare manifestation of LNB.

## Case presentation

**Figure 1:**
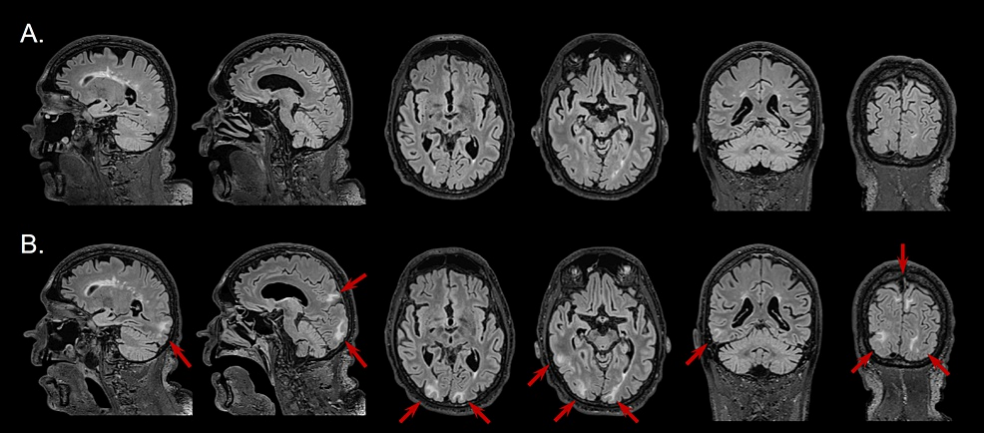
Longitudinal development of changes on T2-flair sequences; day 1 (1A) and day 16 (1B) 1A. Longitudinal development of changes on T2-flair sequences. Day 1 – changes in white matter align with chronic white matter changes of microvascular origin – Fazekas gr.2, no disease-specific changes detected. 1A. Longitudinal development of changes on T2-flair sequences. Day 16 – red arrows point to the changes related to vasogenic edema of subcortical and juxtacortical white matter in the right temporal lobe, bilateral occipital, and mesio-parietal white matter.

**Figure 2:**
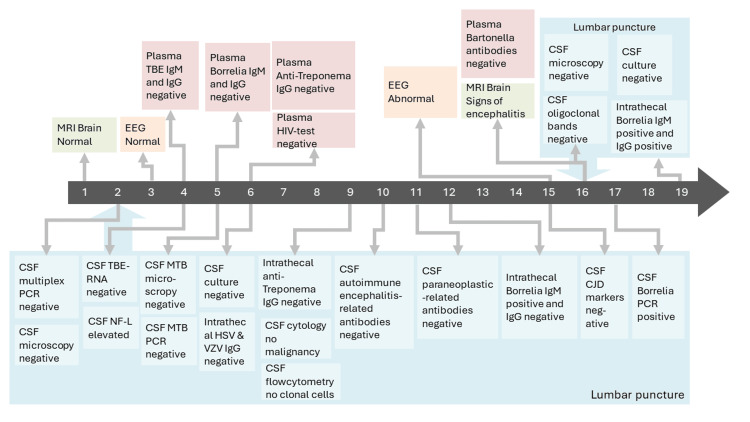
Timeline of test results and imaging Numbers on the dark arrow show days since admission. Gray arrows indicate the day of the result.

A 72-year-old male patient with a medical history of polycythemia vera (treated with hydroxyurea), type 2 diabetes, hypertension, and hypercholesterolemia was hospitalized after three days of confusion and altered mental status in July 2023. Blood samples, computerized tomography (CT), and magnetic resonance imaging (MRI) of the brain showed no relevant abnormalities (Figure 1A). Lumbar puncture revealed CSF with a leucocyte count of 938·106/L (99% mononuclear), protein concentration of 2.33 g/L, and normal glucose level. Initial routine CSF microscopy and multiplex PCR (BioFire FilmArray Meningitis/Encephalitis Panel, Salt Lake City, Utah, USA) did not detect any microorganisms (Figure 2), and the patient started treatment with acyclovir on the suspicion of viral encephalitis. The initial electroencephalogram (EEG) was normal.

Within two days of hospitalization, the patient became somnolent and developed a fever. Ceftriaxone was added to the antiviral treatment, and CSF was further tested for tick-borne encephalitis (TBE), mycobacterium tuberculosis (MTB), intrathecal herpes simplex virus (HSV), varicella zoster (VZV) antibodies, intrathecal Treponema antibodies, autoimmune encephalitis-related antibodies, and paraneoplastic antibodies, which all came out negative (Figure 2). Further, CSF culture was negative, CSF cytology showed no malignancy, and flowcytometry was with no clonal cells. Neurofilament was elevated in CSF. Parallel to this, plasma tests were negative for TBE, Treponema, HIV, and *Borrelia *IgM and IgG (Figure 2).

Eventually, on day 12 of the admission, an intrathecal test for *Borrelia *antibodies (IDEIA Lyme Neuroborreliosis EIA kit, Oxoid, Hampshire, United Kingdom) came out positive for IgM. At this point, however, due to the severity of symptoms and lack of clinical improvement despite ongoing treatment with ceftriaxone, the diagnosis was still not clear-cut. Herpes encephalitis seemed unlikely, and acyclovir treatment was stopped.

**Figure 3:**
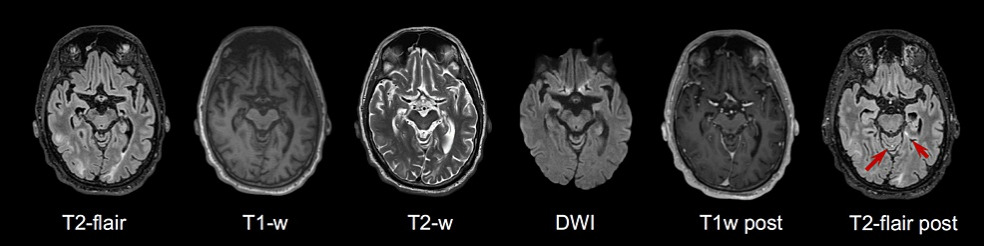
Representative pathological changes on various sequences on MRI of the brain, day 16 Hyperintensities in subcortical and juxtacortical white matter on T2-FLAIR and T2-W with corresponding hypo-intensities on native T1-W image. No diffusion restriction was detected on DWI and ADC maps, and no pathological contrast enhancement was found on postcontrast T1-W image (T1-W post), minimal punctate patchy leptomeningeal enhancement was detected on postcontrast T2-FLAIR images (indicated by arrows).

On day 13 of admission, the patient developed descending consciousness and muscle fasciculations. He was treated with levetiracetam, and the EEG showed signs of a non-specific cerebral lesion. Repeated lumbar puncture on day 16 showed a decreased number of leukocytes (113·106/L, 99% mononuclear), decreased protein (0.73 g/L), and increased glucose (8.4 mmol/L) when compared to the CSF from the first lumbar puncture. A repeated brain MRI on day 16 showed newly developed vasogenic edema, associated with disruption of blood brain barrier, in the bilateral occipital, mesial parietal, and right temporal lobe subcortically, most likely due to encephalitis. No diffusion restriction or definite pathological contrast enhancement in the brain parenchyma was observed neither on the first or second scan while post-contrast T2-flair demonstrated minimal patchy postcontrast leptomeningeal charge leading to the suspicion of incipient leptomeningitis (Figure 1B and Figure 3). Of note, CSF tests for Creutzfeldt-Jakob disease (CJD) and oligoclonal bands were negative. In addition, tests for *Bartonella henselae* and *Bartonella quintana* antibodies in plasma were negative (Figure 2).

On day 17, *Borrelia *DNA was detected in CSF by a PAN-*Borrelia *species in-house real-time polymerase chain reaction (rt-PCR) (Clinical Microbiological Department of Odense University Hospital, Denmark). The sample was subsequently positive in a *Borrelia burgdorferi* sensu lato specific RT-PCR assay as described by Leth et al. [3], thus finally confirming the diagnosis of *Borrelia *encephalitis (Figure 2). Further identification to species level was not possible.

In the following days, the consciousness of the patient gradually improved. He was treated with a total of three weeks of ceftriaxone and discharged with no further treatment. At follow-up one month after discharge, the patient was described as cognitively intact. There was no history of tick bites or rash, but according to the patient’s wife, he had been staying in areas with plenty of ticks and walking in tall grass.

## Discussion

This case shows the diagnostic difficulties for patients with encephalitis due to LNB. Here, we report the first case, to our knowledge, in which both radiological and neurophysiological changes were absent in the initial phase of the disease but developed later during the course.

Encephalitis is, in general, a rare and likely overlooked clinical manifestation of LNB. In a Danish study characterizing 431 patients with a positive *Borrelia *intrathecal antibody index test performed at Odense University Hospital (OUH), the most common clinical symptoms were painful radiculitis (65.9%), cranial nerve palsy (43.4%), and headache (28.3%). Encephalitis was found in 3.7% of all patients. Further, 67% had no *Borrelia*-specific antibodies in the blood at the time of the positive *Borrelia *intrathecal test [4]. As for our case, this emphasizes that a lack of *Borrelia-specific* antibodies in the blood does not rule out neuroborreliosis. Similar results regarding common clinical symptoms and blood antibody response were found in a German study examining the neurological manifestations of LNB leading to hospitalizations [5].

A recent Scandinavian systematic literature review showed that symptoms of encephalitis due to LNB can vary from personality changes and confusion to unconsciousness. A diagnostic delay was observed both from symptom onset to hospitalization (14 days) and until targeted therapy was started (7 days) [6]. Encephalitis prevalence was 3.3% among 1019 screened LNB patients in the Scandinavian cohort described in this article, similar to the study from OUH [4]. In addition, 20.6% of the cases with encephalitis had neuroradiological changes on either CT or MRI [6].

Another recent case report describes a patient with encephalitis due to LNB and parenchymal changes on an MRI of the brain [7]. Even though the MRI was performed two days after admission, the patient had already experienced symptoms for three months. Accordingly, knowledge about changes using CT or MRI over time is still limited and the radiological presentation of the disease on MRI varies and ranges from normal findings to signs of neuritis, meningitis, myelitis, encephalitis, or vasculitis [8]. Of note, no characteristic neuroradiological pattern, such as involvement of the insula, temporal lobe, and limbic system, characteristic for herpes simplex infection, was found in either of the studies, as in our case [8]. Thus, our case emphasizes the use of repeated MRI of the brain in unresolved cases. In the cohort of Scandinavian patients described above, 93.8% of the patients with encephalitis, who had an EEG performed, showed abnormal findings compatible with encephalitis [6]. In contrast, we observed no abnormal EEG changes in the initial phase of our case, but abnormal changes later in the course. As for MRI changes, no specific EEG patterns of LNB are described, as opposed to, for example, Herpes simplex encephalitis, where characteristic changes in the temporal lobes are well-described [9].

A relevant differential diagnosis in this case, is posterior reversible encephalopathy syndrome (PRES), characterized by headache, changes in consciousness, visual changes, seizures, and neuroimaging findings with posterior cerebral white matter edema [10]. The syndrome is associated with hypertension and immunosuppressive therapy, both of which were present in the history of our patient. The MRI findings of vasogenic edema, possibly due to brain-capillary-leak syndrome, are described for both encephalitis and PRES [8,10]. Of note, the immunosuppressive medication was paused on the day of admission where the initial MRI was described as normal. 

However, the finding of intrathecal *Borrelia *antibodies and *Borrelia *DNA, the similarity to other reported cases of *Borrelia *encephalitis, and the clinical course, including the improvement during treatment with ceftriaxone, in our opinion, highly speak in favor of *Borrelia *encephalitis as the final diagnosis. Nevertheless, with the imaging presentations of this case, PRES triggered by the infection cannot be ruled out, and a case of *Borrelia *encephalitis complicated by PRES might be a possible explanation. In a case report describing a child with LNB, LNB is discussed as a possible trigger for PRES [11]. Potential mechanisms might include hypertension due to spirochete involvement in CNS, cytokine-induced vasoconstriction, and secondary to this, the development of vasogenic edema leading to the described clinical picture.

## Conclusions

In conclusion, encephalitis caused by LBN should be considered in the differential diagnosis of unexplained neurological symptoms. The diagnosis might be delayed until positive test results are available; as in our case, 12 days into the admission. Nevertheless, our study clearly illustrates the dynamics of both repeated CSF antibody analysis, imaging, and neurophysiological findings in the case of encephalitis caused by LBN. Therefore, we recommend considering the repetition of tests in diagnostically challenging and unresolved cases, as this might provide additional, valuable diagnostic information. Finally, this case shows an overlap in clinical symptoms and imaging between LNB and PRES.
